# The Impact of Hospitality Work Environment on Employees’ Turnover Intentions During COVID-19 Pandemic: The Mediating Role of Work-Family Conflict

**DOI:** 10.3389/fpsyg.2022.890418

**Published:** 2022-05-19

**Authors:** Ahmed Hassan Abdou, Ayman Ahmed Farag Khalil, Hassan Marzok Elsayed Mahmoud, Mohamed Ahmed Elsaied, Ahmed Anwar Elsaed

**Affiliations:** ^1^Department of Social Studies, College of Arts, King Faisal University, Al-Hofuf, Saudi Arabia; ^2^Department of Hotel Studies, Faculty of Tourism and Hotels, Mansoura University, Mansoura, Egypt; ^3^Department of Geography, College of Arts, Minia University, Minya, Egypt

**Keywords:** work-life conflict, hospitality industry, turnover intention, work-family conflict, COVID-19, coronavirus pandemic

## Abstract

Employees’ turnover intentions and work-family conflict as a result of the hospitality work environment are considered the major global challenges confronted by hospitality organizations, especially in the era of COVID-19. This study aims at identifying the impact of the hospitality work environment on work-family conflict (WFC), as well as turnover intentions and examining the potential mediating role of WFC in the relationship between work environment and turnover intentions, during the COVID-19 pandemic in a sample of three- and four-star resorts in Egypt. A total of 413 resorts employees from Egyptian destinations (Sharm El-Sheikh and Hurghada) participated in the study. The findings of the Structural Equation Modeling (SEM) revealed that the hospitality work environment significantly and positively affects employees’ turnover intentions and WFC. In the context of the mediating role of WFC, results illustrated that WFC significantly partially mediates the relationship between the hospitality work environment and turnover intentions. Upon these findings, the study suggests that to prevent WFC and eliminate turnover intentions among resorts’ employees, an urgent need to create a better work environment is vitally important. limitations and future research directions have been discussed.

## Introduction

In comparison to other sectors of the world economy, the hospitality industry is considered one of the fastest developing sectors all over the world (International Labour Organisation [ILO], 2001). Globally, this industry creates employment that requires various skills and facilitates rapid access to a diverse workforce including women, youth, and migrant workers (Kusluvan et al., 2010). Despite this fact, it is constantly growing, the hospitality industry faces its unique workforce challenge where employees’ turnover is extremely high (Baum, 2008; Davidson et al., 2010). Building and maintaining a sustainable workforce and eliminating turnover intentions of organizational employees have turned into one of the foremost global issues confronted by hospitality organizations (Yam et al., 2018; Murray and Holmes, 2021). Barron (2008) illustrated that the reputation of the hospitality industry as a key means of permanent employment is not good and holds a negative image for offering limited chances for career development. Long, irregular, and unsociable working hours, work under pressure, irregular holidays, heavy workloads, family-unfriendly work shifts, low job stability and security, relatively low salaries, and seasonality are some of the common characteristics of the hospitality industry, which represent the most predictors of employees’ turnover intentions (Cho et al., 2009; Brien et al., 2015). Moreover, such conditions at work result in anxiety in the employees having family responsibilities, especially women that care about children, and the elderly and carry out most of the household chores (International Labour Organisation [ILO], 2001).

In addition to the poor or unsuitable work conditions, WFC might be perceived as one of the main predictors of employees’ turnover intentions (Karatepe and Baddar, 2006). WFC occurs in both directions (work-family conflict and family-work conflict), as work-life can influence family life and vice versa (Tetrick and Buffardi, 2006). WFC arises once the work roles of employees hamper their capability to fulfill their family roles. Meanwhile, family-work conflict (FWC) exists once the family roles of employees hamper their capability of meeting their work needs (Mesmer-Magnus and Viswesvaran, 2005). As a result of the nature of business 24/7 and the culture of “face-time,” the hospitality industry is commonly perceived as hectic, and the conflict between employees’ work and family will be a prominent characteristic of the place of work (O’Neill and Xiao, 2010).

Numerous studies illustrated that WFC has significant and positive effects on turnover intentions (Boyar et al., 2003), burnout and employees’ dissatisfaction (Bruck et al., 2002; Barriga Medina et al., 2021), and adverse impacts on the well-being and health of the individuals (Huang et al., 2019), are linked to the symptoms of physical health and psychological anxiety (O’Driscoll et al., 2004), and are negatively related to organizational commitment (Cao et al., 2020). Amstad et al. (2011) reached through their meta-analysis the conclusion that WFC is negatively associated with life, marital, and family satisfaction.

The interrelationship between work environment, turnover intentions, and WFC has been tackled from diverse perspectives. For instance, Mathew and Vijayalakshmi (2017) found out that the existence of an affirmative work environment represents the foremost component of the total reward strategy of any organization. On the contrary, an unsupportive working environment promotes the negative job attitudes of people toward their organization, decreases employees’ retention, and supports turnover intention (Poulston, 2009). Markey et al. (2012) stated that the work environment is the foremost element impacting the turnover intentions of employees or their decision of staying in the organization. A study conducted by Hoque (1999) on a sample of employees in the United Kingdom hospitality industry revealed that lack of job security, extended working hours, high demand for coordinating with others, and irregular shifts are reasons for women’s WFC, and they may lead to employees’ turnover. In the hospitality industry, one of the foremost significant barriers to operative human resource management is WFC (Tromp and Blomme, 2012). In the health sector, the findings of an experimental study including 250 doctors employed in governmental Pakistani hospitals aimed to identify the impact of WFC on turnover intentions declared that WFC significantly and positively affects turnover intentions (β = 0.585, *p* < 0.01) (Asghar et al., 2018). In 2017, Rubel et al. studied the mediating role of WFC on the association amid the three extents of role stressors (role conflict, role ambiguity, and role overload) and turnover intention of 365 supervisory staff employed in the ready-made garment (RMG) industry of Bangladesh. The findings of the study revealed that role stressors increase the WFC of the employees leading to turnover intention. Additionally, WFC significantly mediated the relationship between turnover intentions and role stressors.

As a result of the COVID-19 pandemic, the hospitality work environment has dramatically changed. After the healthcare practitioners, the next high-risk occupation was a variety of job positions in the tourism and hospitality sector, marked as particularly vulnerable to the risk of contracting the disease (Chinazzi et al., 2020). The global panic caused by the COVID-19 outbreak increased anxiety, stress, depression, and frustration, especially among hospitality service providers (Aguiar-Quintana et al., 2021). The study conducted by McCartney et al. (2022) to examine the impacts of the COVID-19-induced lockdown on employees’ turnover intentions for the hospitality retail sector revealed that the investigated respondents perceived a lower level of workload and pay dimension (including, the working hours of current job fit with private family life, workload matches salary, and the workload of the current job is fair). Workload and pay dimensions had the greatest influence on employees’ turnover intentions. Because of mobility restrictions and lockdowns during the COVID-19 pandemic, workplace interactions were limited or completely prevented (McCartney et al., 2022). Furthermore, as a result of the COVID-19 outbreak, the lack of financial resources was the dominant feature in most hotels, which led to a reduction in the employees’ salaries (Bajrami et al., 2021). Staff was unable to receive additional sales incentive payments and bonuses, relying on base pay from the hotel (McCartney et al., 2022).

Despite previous studies have examined work environment, WFC, and turnover intentions, no research has explored the interrelationships between work environment, WFC, and turnover intentions specifically in hospitality contexts during the COVID-19 pandemic. To the best of our knowledge, this study is the first in hospitality literature that investigates the interrelationships between the hospitality work environment, work-family conflict, and turnover intention during the COVID-19 pandemic specifically in the resort context. This study is significant to determine the perceptions of resorts’ employees toward the hospitality working environment during the COVID-19 pandemic and determine its direct impacts on work-family conflict, as well as employees’ turnover intentions. It is also important to examine the direct effect of work-family conflict with its three dimensions (time-based conflict, strain-based conflict, and behavior-based conflict) on employees’ turnover intentions. Furthermore, the study contributes to identifying the indirect relationship between the hospitality work environment and turnover intentions through WFC during the COVID-19 pandemic, which has not been addressed before. Moreover, the study findings will contribute to identifying the common factors affecting turnover intentions during the COVID-19 pandemic. The findings of the study are expected to be able to find a solution to the problem of increasing turnover intention and balancing work-family relationships during the COVID-19 pandemic. Therefore, this study aims at identifying the impact of the hospitality work environment on WFC, and turnover intentions, and determine the potential role of WFC as a mediator in the relationship between work environment and turnover intentions during the COVID-19 pandemic in a sample of three- and four-star resorts in Egyptian destinations.

In our study, the theory of planned behavior (TPB) was employed as a guide for the study’s theoretical model to better understand the antecedents of hospitality employees’ turnover intentions. TPB is a psychological theory that elucidates the psychological phenomena of human behavioral intention (Ajzen, 1991). According to the TPB theory, an individual’s behavioral intentions are shaped by three essential characteristics, as follows; the person’s favorable or unfavorable attitude toward performing a behavior; subjective norms and perceived social pressure to perform the intention or not; and perceived behavioral control, i.e., the opportunities and resources available to the individual will influence the likelihood of the behavior occurring (Ajzen, 1991; McCartney et al., 2022). Zhao et al. (2021) utilized TPB to examine the relationships among resilience, job satisfaction, social support, and turnover intention of nurses. In the hospitality industry context, McCartney et al. (2022) employ the TPB to investigate the impact of the co-worker relationship, company support, and workload and pay on hospitality retail employees’ turnover intention during the COVID-19 pandemic. In this study, we assume that the perceived hospitality work environment as well as WFC including its three dimensions (time-based conflict, strain-based conflict, and behavior-based conflict) act as key predictors of resorts’ employees’ turnover intentions.

The present study focuses on resorts’ employees for different reasons. Firstly, most of the resorts are located in remote areas that don’t have a ready labor market. Secondly, seasonality is the dominant feature of Egyptian resort hotels. The irregular and occasional demand for labor affects negatively employees’ retention and commitment. Thirdly, the majority of employees come from distant areas and by prearrangements. They stay on duty for a long time and may remain in their workplace for the season. Consequently, resort management should house, feed, clothe, and cares about the employees who stay for a long time away from their homes. All the previous resorts’ characteristics could affect WFC and turnover intentions.

This study has been split up into six sections. Section “Introduction” briefly discusses the introduction. Section “Theoretical Background and Hypothesis Development” deliberates on the theoretical background concerning the association among work environment, turnover intentions, and WFC in the hospitality industry. Section 3, “Materials and Methods,” describes the measures, sampling, and data collection methods adopted in this study. Moreover, the results of the study are presented and analyzed in Section 4, “Results.” Section “Discussion and Implications” shows how the results were discussed. Furthermore, theoretical, and practical implications have been addressed. Limitations and suggested guidelines for further research have also been discussed in Section “Limitations of the Study.”

## Theoretical Background and Hypothesis Development

### Hospitality Industry in the Era of COVID-19 Pandemic

Since COVID-19’s appearance, firms in many industries worldwide experienced a sudden, temporary, and sharp reduction in revenue (Tran et al., 2020; Abdou and Shehata, 2021). The coronavirus pandemic upended work, family, and social life (Schieman et al., 2021). Coronavirus outbreaks had a major impact on global public health, as well as the supply and value chains of various industries (Lu and Lin, 2021). One of the domestic industries severely affected, for example in Egypt, during the pandemic was the hospitality industry (Salem et al., 2021). The business is decreasing day by day due to the COVID-19 spread (AlZgool et al., 2021). Many hospitality businesses were forced to temporarily close due to the measures taken to mitigate the spread of the virus. These measures included physical/social distancing, community lockdowns, orders to stay home, and travel and mobility restrictions (Aharon et al., 2021).

As the pandemic continues, numerous studies and reports have concluded that the COVID-19 pandemic negatively affects the hospitality and hotel business. For example, in 2019, the global market size of the hotel and resort industry was at its peak at 1.47 trillion U.S. dollars. In 2020, owing to the coronavirus pandemic, the market size declined to 610 billion U.S. dollars (Statista, 2021a). In May 2020, the hotel industry in Europe has taken a bad hit, where the hotel occupancy rate reached 13.3 percent, down from 82.3 percent in the previous year (Statista, 2021b). Furthermore, the revenue per available room (RevPAR) achieved the foremost dramatic impact of the virus, dropping to 11.35 U.S. dollars compared to the previous year (85.33 U.S. dollars) (Statista, 2021c). The study carried out by Rodríguez-Antón and Alonso-Almeida (2020) illustrated that the performance of Spain’s hospitality industry has been dramatically affected by the coronavirus pandemic. Specifically, they reported that the total number of hotel overnight stays in Spain decreased from 184.7 million in 2019 to nearly 46.4 million in the first 7 months of 2020. In China, Hao et al. (2020) assured that the hospitality industry suffered a sudden decline in hotel occupancy rates and a loss of more than US$9 billion in revenue in January and February 2020 for an average period of 27 days. The hotel occupancy percentage dropped from around 70 to 8% and nearly 74% of the Chinese hotels were closed. In March 2020, the Egyptian hospitality sector started to dramatically decline, nearly 70 to 80% of hotel bookings have been cancelled (Egyptian Center for Economic Studies, 2020). Breisinger et al. (2020a) estimated that the absence of tourists alone may cause monthly losses of $1.5 billion. In the case of a fast recovery scenario, Breisinger et al. (2020b) projected that hotels loss was reduced by only 50 percent.

Employment in the hospitality industry suffered greatly from the pandemic. According to the United States Bureau of Labor Statistics, the hospitality industry lost 3.5 million jobs during the COVID-19 pandemic accounting for more than a third of all unemployed persons in the United States. Only in August 2021, did about 892,000 hospitality employees quit their job (Croes et al., 2021).

Responses to the COVID-19 epidemic, some hospitality businesses have employed a series of approaches to address the negative concerns. A study conducted by Croes et al. (2021) aimed to investigate the impacts of COVID-19 on hospitality employees and identify the support they received from their operations during the COVID-19 pandemic showed that some drastic measures were taken to avoid business closures such as; reducing shifts/hours (41%), concerned for health and safety due to COVID-19 (33%), having insufficient money to meet their expenses (31%), furloughed (22%), and laying-off (21%). Regarding support received during the pandemic, a total of 44% of the investigated participants mentioned that they received unemployment wages, 24% delayed bill payments, 21% received nutritional assistance (i.e., food stamps), 15% received aid in the form of medical care, and 20% received housing and other financial aid. Salem et al. (2021) suggested some measures that employers should consider to maintain and retain the hospitality employees during the COVID-19 pandemic such as: providing paid vacations, reducing salaries and benefits of employees instead of laying them off, redeploying staff to new positions or lines within the business, caring of employees’ health through providing equipment to detect viruses and infectious diseases, providing personal protective equipment (masks, gloves), and increasing their health awareness of signs and symptoms of COVID-19 through a series of health information sessions.

### Work Environment and Employees’ Turnover Intentions

The work environment is termed as the non-financial factor that creates a conducive atmosphere where employees can accomplish their work (Chao, 2008). The work environment consists of various wide-ranging hectic and helpful features of the work milieu including work motivators, work stressors, physical environments, and relationships with colleagues besides various supervision and management behaviors (Billings and Moos, 1982). Furthermore, the work environment could be categorized into two sections: (1) physical environment, and (2) non-physical environment (Adewale et al., 2020). Physical environment, such as temperature, lighting, design, and equipment, and all physical aspects that affect directly and indirectly employees’ attitude and behavior. Non-physical work environment refers to the form of a harmonious work environment, which include the relationships between superiors and subordinates and the relationship between fellow employees (Sitepu et al., 2020). In this context, a good atmosphere, encompassing attractive, clean, stimulating, and caring conditions, positively affects the employees’ commitment and retention (AlBattat et al., 2014). A positive work atmosphere extremely contributes to attracting and maintaining the skillful employees of any organization (Amin and Akbar, 2013; Msengeti and Obwogi, 2015).

As mentioned by Akgunduz and Eryilmaz (2018), an employees’ turnover intention refers to their mindfulness or thoughts regarding leaving their current jobs. Blomme et al. (2008) recognized the intent of leaving as a plan of an employee to resign from the current profession and search for another career soon. It is considered vital evidence of genuine voluntary turnover (Jawahar and Hemmasi, 2006). A lot of research studies regarding the hospitality context have explored turnover intention and identified the factors that are capable of impacting employees’ turnover intention (i.e., Blomme et al., 2008; Lam and Chen, 2012). In their meta-analysis, Park and Min (2020) studied 35 previous predictors of turnover intention related to the hospitality industry. Based on 391 correlations found from 144 independent research studies, they concluded that role stressors/inter-role conflicts, job strains, and work attitudes exhibited comparatively significant impacts on turnover intention.

Several studies focused on the relationship between the work environment and employees’ turnover intentions and turnover rate from different perspectives. Zeytinoglu et al. (2004) revealed that short and split-shifts, lack of job security, irregular working hours, low wages, and the necessity of maintaining numerous careers, result in anxiety, higher turnover intention, and diverse conflicts in the workplace. Kuria et al. (2012) revealed that the unconducive work settings, extended working hours involving nominal wages, and poor training provided to the employees resulted in the development of the employees’ work-related stress, reducing employees’ organizational commitment, and increasing turnover intentions. AlBattat et al. (2014) illustrated that poor training, unacceptable working conditions, and low wages are capable of leading to higher employees’ turnover.

Workplace flexibility and supervisors’/managers’ behavior and their impact on the behavior and attitude of employees have drawn significant academic interest in hospitality research. Workplace flexibility and supervisors’ supportive behavior promote employees’ perspectives to change relationships with their organizations, which in turn affect their organizational commitment and turnover intention (Panaccio and Vandenberghe, 2009; Cheng et al., 2013; Jung and Yoon, 2021). Kim et al. (2015) examined how mentoring activity including three foremost purposes (role modeling, psychosocial assistance, and career development) might improve job attitudes and role stress and lessen turnover intentions amongst staff in the hospitality industry. The study also explained that the psychosocial assistance function displayed a noteworthy association with all variables of the study (i.e., an optimistic impact on organizational commitment and job satisfaction, but a negative impact on turnover intention, role ambiguity, and role conflict). Organizational justice encompassing its three dimensions (interactional, procedural, and distributive justice) is directly associated with turnover intentions (Nadiri and Tanova, 2010). In this study, we seek to understand the impact of the hospitality work environment (physical and non-physical) including working conditions and characteristics, supervisors’ social support, supervisors’ organizational justice, interrelationship with colleagues, working under pressure, and the flexibility of the workplace on employees’ turnover intentions. Upon the previous literature, we assumed that:

H_1_: The hospitality work environment significantly affects employees’ turnover intentions.

### The Relationship Between Work Environment and WFC

Work-family conflict (WFC) could be defined as “A form of inter-role conflict in which the role pressures from the work and family domains are mutually incompatible in some respect” (Greenhaus and Beutell, 1985; p. 77). Greenhaus and Beutell (1985) categorized WFC into three dimensions as follows: time-based conflict, behavior-based conflict, and strain-based conflict. Time-based conflict exists when the time required to achieve one role prevents to fulfillment of activities in the other role (i.e., an employee who must spend more time in his/her work or work on holidays, as a result, cannot tend to family needs or attend a family gathering). Behavior-based conflict takes place once persons are incapable of adjusting their behaviors related to diverse roles. Behaviors which are suitable for one role may be inconsistent with another one. For example, the methods of handling guest complaints in the workplace may not be appropriate for family members (Magnini, 2009). As a final point, strain-based conflict ensues once an individual feels anxiety due to one role which consequently leads to unsuccessful participation in another role. Work overload, exhaustion, and tensions from work conditions negatively affect the quality of employees’ home life stability (Li et al., 2021).

The impact of WFC has been tackled from diverse perspectives. Various studies illustrated that exposure to this conflict for a long time may yield negative psychological and behavioral outcomes. Long term exposure to WFC increases job stress (Krisor et al., 2015), reduces job satisfaction (Carlson and Kacmar, 2000; Choi and Kim, 2012), predicts turnover intention (Karatepe and Uludag, 2008), positively related to job burnout (Karatepe et al., 2010), and negatively links to employees’ performance (Karatepe, 2013) and life satisfaction (Karakose et al., 2021).

Exposure to work stressors, such as unpredictable schedule requirements (Presser, 2003), unsociable working hours, including evening and night shifts (Henly et al., 2006), and minimal control over working hours (Swanberg et al., 2008), are positively related to higher WFC. Conversely, in their study conducted on 247 highly educated employees (Graduates of the Hotel school The Hague, Netherlands), Blomme et al. (2010) illustrated that workplace flexibility and organizational support affect negatively WFC, especially for female employees. They recommended that offering flexible working hours besides sustaining a good organizational environment should be considered to lessen WFC and retain highly educated staff. Upon that, we hypothesized that:

H_2_: The hospitality work environment significantly affects WFC.

### WFC and Employees’ Turnover Intentions

Several theoretical frameworks have dealt with the association between turnover intentions and WFC in different sectors. A study involving 150 workers from five diverse industry areas (telecom, banking, insurance, IT software development, and IT-enabled services) aimed at exploring the strength of relationships among WFC of employees and their intention of leaving the organization revealed that WFC is significantly predicting turnover intentions. Time-based as well as strain-based conflict exhibited the highest correlations with turnover intention, showing dissimilarities across diverse industry sectors (Aboobaker et al., 2017). The findings of an empirical study conducted on employees of the Pakistani banking sector revealed a positive and significant impact of WFC on turnover intentions (Alsam et al., 2013). Moreover, Azhar et al. (2016) aimed in their study to explore the association between WFC and turnover intention amongst the academicians at Malaysian higher academic institutions. The findings of the study illustrated that WFC has a positive and significant interrelationship with turnover intention (*r* = 0.262, *p* < 0.01).

In the hotel sector in Quebec Province (Canada), Mansour and Tremblay (2018) examined the impacts of WFC on job burnout, job anxiety, and intention of leaving the organization in a study involving 258 hotel employees. The study findings illustrated that WFC is the foremost predictor of employees’ intention to leave. In 2018, a comparative study has been conducted to explore the association between turnover intentions and WFC among hotel staff in both China and the United States (Chen et al., 2018). The study’s results confirmed that WFC is positively associated with the turnover intentions of hotels’ employees. However, WFC was more strongly associated with turnover intentions among males than females. The association is also more resilient in the female staff of the Chinese hotel than their United States corresponding person. Hence, we hypothesized that:

H_3_: WFC significantly affects employees’ turnover intentions.

### The Mediating Role of WFC on the Relationship Between Work Environment and Turnover Intentions

Despite the findings of the previous studies (i.e., Presser, 2003; Zeytinoglu et al., 2004; Chen et al., 2018), which confirmed that the work environment significantly affects WFC and turnover intentions, and WFC significantly predict turnover intentions, the role of WFC as a mediator in the relationship between hospitality work environment and turnover intentions has not been examined. There is evidence that WFC could mediate the relationship between the hospitality work environment and employees’ turnover intentions. Thus, we hypothesized that:

H_4_: WFC significantly mediates the relationship between the hospitality work environment and employees’ turnover intentions.

**FIGURE 1:**
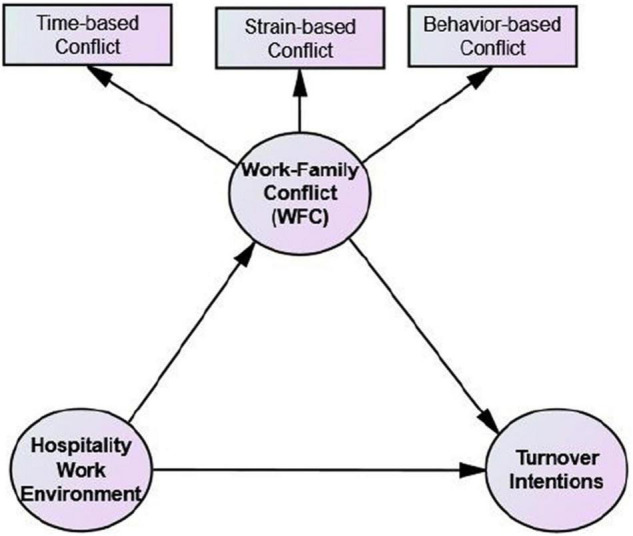
The conceptual model of research.

The conceptual model of research is presented in Figure 1.

## Materials and Methods

### Measures and Instrument Development

Responses to COVID-19 pandemic restrictions (i.e., physical and social distancing), the main data collection in this study was a web-based questionnaire survey. Recently, internet-based data collection technique such as online questionnaire has become more popular, especially in quantitative research methodology (Van Selm and Jankowski, 2006). The questionnaire was developed based on a comprehensive appraisal of the study literature to identify valid and frequently used measures. The questionnaire consisted of four sections. The first section took account of the participants’ demographic data, which encompassed age, gender, level of education, level of position, and marital status. The second, third, and fourth sections were intended to reveal the participants’ perceptions of the hospitality work environment, WFC, and turnover intentions, respectively.

Hospitality work environment scales utilized by Kusluvan (2003) and Arnoux-Nicolas et al. (2016), were adapted and applied to identify the perceptions of the investigated participants toward the hospitality work environment by using a five-point Likert scale having the range from 1 = strongly disagree, to 5 = strongly agree. The scale is composed of nine items (i.e., long, irregular, and unsociable working hours, and a hostile work climate among colleagues). A higher value of the average score reflects a more negative work environment provided by the investigated resorts. The internal consistency reliability (Cronbach’s alpha) for the hospitality work environment scale was 0.925.

About WFC, a multidimensional WFC scale was utilized and validated in different studies (i.e., Carlson et al., 2000; Vieira et al., 2014; Wang et al., 2017; Shin and Jeong, 2020) was used to measure WFC among the investigated participants. A most significant contemplation while utilizing this scale is that this scale gives due consideration to the bi-directional relationship of the FWC/WFC. In this study, we only focus on the WFC scale. The scale is categorized into three dimensions, each one has three items, as follows: (a) time-based conflict (i.e., “My work time keeps me away from the activities of my family more than I would desire”), (b) strain-based conflict (i.e., “After getting home from work, I am frequently too fatigued to take part in activities/responsibilities of the family”), and (c) behavior-based conflict (i.e., “Behavior which is necessary and effective for me at work would be counterproductive at home”). A higher mean score indicates a greater WFC. The internal consistency reliability (Cronbach’s alpha) for the WFC scale was 0.895.

The turnover intentions were evaluated by using a modified three-item measure scale based on Blomme et al. (2010) and Jung et al. (2021). These items are: (1) “Currently, I am really thinking of resigning from my present job in the resort, (2) Probably, I will try to find a new profession within a year, and (3) If I have an option to choose again, I will opt to work in another job. The response rate was also calculated by using a five-point Likert scale ranging from 1 = strongly disagree, to 5 = strongly agree. A higher value of the average score reflects a higher intention to leave. The internal consistency reliability (Cronbach’s alpha) regarding the turnover intention scale was 0.893.

The survey was initially prepared in English and was then translated into participants’ native Arabic language and then reverse translated from Arabic to English to confirm that there were no differences in meaning. To assure that the study instrument measures the constructs that it sets out to measure, the questionnaire’s face validity was tested by five hospitality academics, who were asked to assess the content of the questionnaire form and provide any feedback. Furthermore, a pilot study has been conducted on a sample of (30) resorts’ employees, who are not included in the study’s main sample, to examine the feasibility of the questionnaire by testing whether the questionnaire was comprehensible and appropriate and whether the questions were well-defined, clearly understood and presented consistently. Upon the participants’ comments, a modification was made to the wordings of some statements, and even some statements were also re-ordered.

### Sampling and Data Collecting

As mentioned previously, the key aim of the present study is to identify the impact of the hospitality work environment on WFC, and turnover intentions, and to explore the potential mediating role of WFC in the relationship between work environment and turnover intentions during the COVID-19 pandemic in a sample of three- and four-star resorts in Egyptian destinations (Sharm El-sheikh and Hurghada). For achieving this objective, an online questionnaire was developed and forwarded to chosen employees to identify their perceptions of study constructs (work environment, WFC, and turnover intentions). The research team, through their relationships with resorts’ human resources managers and employees in Egyptian destinations, invite them to take part in the field study. The convenience and snowball sampling techniques were used for the study.

As a result of the COVID-19 pandemic, which led to the layoff of a large number of employees in the hospitality industry, there was a shortage in the number of participants for this study. Consequently, to overcome this critical issue, the study sample was firstly recruited by using the convenience sample method “a type of non-probability sampling, in which people are sampled simply because they are convenient sources of data for researchers” (Lavrakas, 2008). Secondly, the online snowball sampling technique, which was widely used in hospitality research during the pandemic to reduce the risk of COVID-19 transmission was also employed (Kang et al., 2021; Üngüren and Yildiz, 2021; McCartney et al., 2022). The potential participants were asked to complete the questionnaire form and to forward the invitation to their colleagues who are still on the job.

The questionnaire was administrated *via* an electronic Google form and the link was sent online to participants *via* WhatsApp and email. A welcome message along with detailed information about the study purpose has been sent. Participants were told that participation in the study is voluntary. They were kindly asked to check the appropriate answer on a scale from 1 to 5, where 1 = strongly disagree and 5 = strongly agree. Finally, after completing the survey they were asked to submit it. Data collection spanned almost 2 months (November–December 2021).

The appropriate sample size according to the recommendation of Hair et al. (2010) and Kock and Hadaya (2018) was decided. They recommended calculating the appropriate sample size based on the number of the investigated variables. The minimum ratio (variable: sample = 1:10) is acceptable. Consequently, the minimum sample size required for this study was 210 participants, where the total variables under investigation are 21 variables.

To reduce the threat of common method variance/bias (CMV), it was assured to the investigated participants that collected data would be kept anonymous, confidential, and would be used only for the research purpose. They were asked to answer the questions with complete honesty where there are no correct or incorrect responses. Moreover, Harman’s single-factor test as a widespread and simple statistical tool that discovers CMV was used (Rodríguez-Ardura and Meseguer-Artola, 2020).

The valid responses from the investigated participants were 413, most of them (*N* = 294, 71.2%) were males and 28.8% (*N* = 119) were females. Regarding the ages of the participants, participants having an average age of 25 to 35 years were found to be in the higher category (*N* = 257, 62.2%). In the context of the educational level, more than half (*N* = 214, 51.8%) had a bachelor’s degree. A higher percentage of the investigated participants represent entry-level positions (*N* = 297, 71.9%). Married employees constituting 62.7% (*N* = 259).

### Data Analysis

The collected data were analyzed by using SPSS v. 22 and Amos v. 26. For representing the demographic data of the investigated respondents and identifying their perceptions toward study constructs, descriptive statistics, encompassing mean, standard deviation (SD), frequencies, and percentage, were utilized. Common method variance (CMV) was examined through Harman’s single factor test. The validity and reliability of measurement items were confirmed by reliability analysis (Cronbach’s alpha) and confirmatory factor analysis (CFA). The average variance extracted (AVE), composite reliability (CR), and Maximum Shared Variance (MSV) were calculated for the confirmation of validity. Discriminant validity based on Fornell–Larcker criterion was also examined. Finally, structural equation modeling (SEM) was employed for determining the direction, as well as interrelationships between study hypotheses.

## Results

### Descriptive Statistics

Table 1 shows the mean and SD of each variable related to the constructs of the study. About the hospitality work environment, the investigated respondents agreed and strongly agreed on most of the investigated items, where the average mean ranged from 3.59 to 4.46. They ranked (Irregular and unsocial working hours) as the highest negative work environment attribute (M = 4.46, S. D. = 0.831). Furthermore, participants perceived a higher level of WFC, as well as turnover intention. The mean values of items under WFC and turnover intention ranged from 4.23 to 4.45 and 4.19 to 4.25, respectively. The investigated respondents showed that working in resorts keeps the employee from their family activities more than it should be, with an average mean of 4.45 (S.D. = 0.754) as the highest item related to WFC. However, they ranked (“Currently, I am really thinking of resigning from my present job in the resort”) with an average mean of 4.25 (SD = 0.934) as the highest items related to employees’ turnover intentions.

**TABLE 1:** Descriptive statistics, reliability, and confirmatory factor analysis properties.

Construct	M^1^ (S.D.)^2^	Std. Loading (CFA)^3^	*t*-value	CR^4^	Cronbach’s alpha	AVE^5^	MSV^6^
*(1) Hospitality Work Environment*				*0.927*	*0.925*	*0.586*	*0.279*
*We1*: Working conditions (i.e., equipment, lighting, ventilation…etc.) is poor and inappropriate.	4.23 (0.955)	0.745	Fixed				
*We2*: Seasonality and job insecurity (difficulty to get a stable job in resorts).	3.98 (1.136)	0.742	15.338***				
*We3*: Irregular and unsocial working hours, including shifts and work at night and weekends.	4.46 (0.831)	0.611	12.42***				
*We4*: Shortage of staff which results in a work overload.	4.02 (1.132)	0.857	18.025***				
*We5*: Lack of social support when needed.	4.12 (0.990)	0.732	15.098***				
*We6*: A hostile work climate among colleagues (poor co-worker attitudes).	4.01 (1.212)	0.752	15.569***				
*We7*: Absence of employee empowerment and participation.	3.73 (1.296)	0.825	17.257***				
*We8*: Unfairly and inequality from managers/supervisors when dealing with staff.	3.59 (1.369)	0.845	17.734***				
*We9*: Working under pressure with different cultures and nationalities.	4.13 (1.045)	0.750	15.530***				
*(2) Work-Family Conflict (WFC)*				*0.861*	*0.895*	*0.674*	*0.279*
*(a) Time-based Conflict*							
*Wfc1*: working in resorts keep employees from their family activities more than it should be.	4.45 (0.754)	0.824	Fixed				
*Wfc2*: The long working hours/periods keep employees from participating equally in household responsibilities and activities.	4.41 (0.772)	0.848	18.878***				
*Wfc3*: Employees have to miss their family activities due to the amount of time which should spend on work.	4.42 (0.763)	0.829	18.464***				
*(b) Strain-based Conflict*							
*Wfc4*: Working in resorts is a frazzle to participate in family activities/responsibilities.	4.44 (0.727)	0.817	Fixed				
*Wfc5*: Employees are so emotionally drained when they get home from work that it prevents them from contributing to their family.	4.42 (0.754)	0.773	15.766***				
*Wfc6*: Due to the work pressures, employees transfer their strain to their family	4.27 (0.910)	0.732	14.918***				
*(c) Behavior-based Conflict*							
*Wfc7*: The problem-solving behaviors used in the job are not effective in resolving problems at home.	4.23 (0.886)	0.761	Fixed				
*Wfc8*: The commitment at work (i.e., shifting the work periods) would be counterproductive at home.	4.38 (0.836)	0.860	17.369***				
*Wfc9*: Behavior that is effective and necessary for work does not help the employees to be better parents and spouses.	4.34 (0.854)	0.841	17.056***				
*(3) Turnover Intention*				*0.897*	*0.893*	*0.745*	*0.249*
*Ti1*: Currently, I am really thinking of resigning from my present job in the resort.	4.25 (0.934)	0.763	Fixed				
*Ti2*: Probably, I will try to find a new job next year or less.	4.19 (0.909)	0.918	19.453***				
*Ti3*: If I have an option to choose again, I will opt to work in another job.	4.22 (0.904)	0.900	24.98***				

M^1^ = Mean, (S.D.)^2^ = Standard Deviation, Std. Loading, (CFA)^3^ = Standardized Factor Loading, CR^4^ = Composite Reliability, AVE^5^ = Average Variance Extracted, MSV^6^ = Maximum shared variance. Model fit; x^2^ = 509.041 (df = 183) p < 0.001, x^2^/df = 2.782, Goodness of Fit Index (GFI) = 0.901, Comparative Fit Index (CFI) (0.942, Normed Fit Index (NFI) (0.912, Root Mean Square Residual (RMR) (0.048, Incremental Fit Index (IFI) (0.942, Relative Fit Index (RFI) (0.899, Root-Mean Square Error of Approximation (RMSEA) (0.066, ***p < 0.001.

### Measurement Model

As mentioned previously the study data were collected by using a web-based questionnaire. Therefore, a common method of variance/bias (CMV) was first identified using Harman’s single-factor test (Podsakoff et al., 2003). As a result, one component was found to account for only 38.7% (smaller than 50%) of the variance which reveals that CMV does not represent a concern.

**FIGURE 2:**
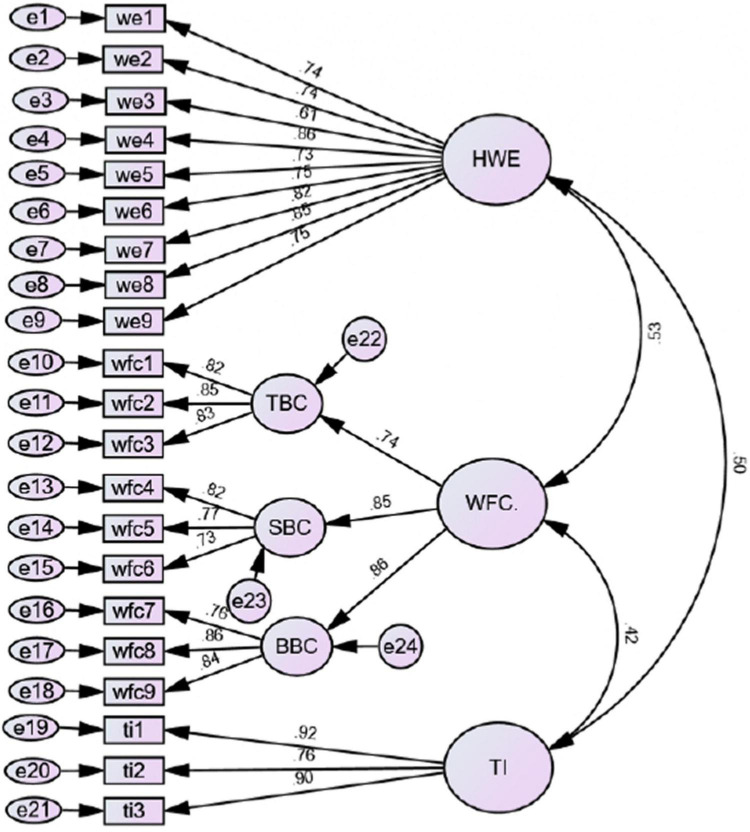
Confirmatory factor analysis.

To explore the validity and reliability of the study constructs, CFA using maximum Likelihood was conducted (see Figure 2). As presented in Table 1, values of Cronbach’s alpha and composite reliability (CR) of all latent variables exceed the suggested threshold of 0.80 (Hair et al., 2013), specifying acceptable internal reliability. Construct validity was also examined by utilizing convergent and discriminant validities (Chin et al., 1997). Convergent validity necessitates a factor loading of not less than 0.50 and an average variance extracted (AVE) above 0.50 (Duckworth and Kern, 2011). The factor loading of all study objects is higher than 0.50 and the AVE of each construct was above 0.50, ranging from 0.586 to 0.745, which indicates that convergent validity has been achieved. Based on the criterion of Fornell-Larcker, the constructs’ discriminant validity requires the square root of AVE of every construct to be greater than its correlation with another construct. Data in Table 2 illustrated that the AVE square root of all constructs is higher than their correlations with other ones. Moreover, the maximum shared value (MSV) scores were less than the AVE of each construct (Hair et al., 2010). Results in Tables 1, 2 confirm the adequacy of discriminant validity of study constructs (Henseler et al., 2015).

**TABLE 2:** Discriminant validity based on Fornell–Larcker criterion.

Construct	1	2	3
1- Work Environment	**0.766**		
2- WFC	0.528	**0.821**	
3- Turnover Intentions	0.499	0.421	**0.863**

*Bold diagonal numbers represent the square root of AVE’s study constructs.*

The fit of the study’s model was acceptable; x^2^ = 509.041 (df = 183) *p* < 0.001, x^2^/df = 2.782, Goodness of Fit Index (GFI) = 0.901, Comparative Fit Index (CFI) = 0.942, Normed Fit Index (NFI) = 0.912, Root Mean Square Residual (RMR) = 0.048, Incremental Fit Index (IFI) = 0.942, Relative Fit Index (RFI) = 0.899, Root-Mean Square Error of Approximation (RMSEA) = 0.066.

### Structural Equation Modeling

**FIGURE 3:**
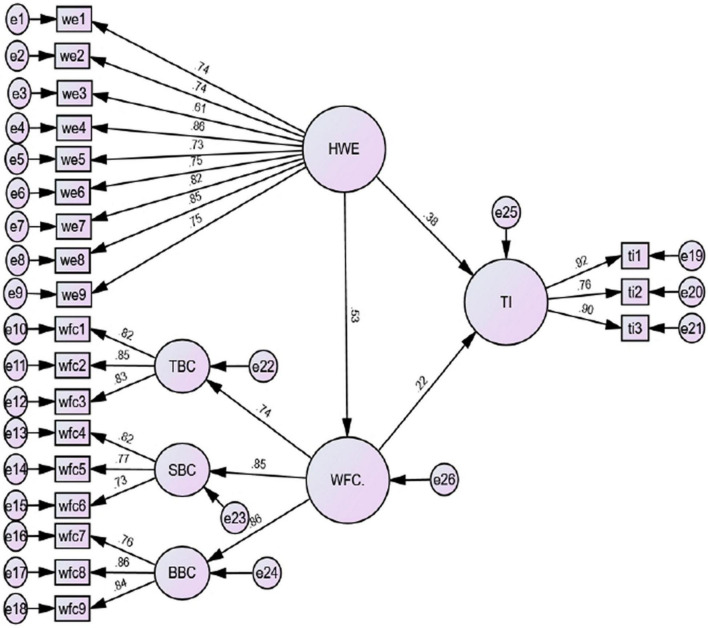
The structural model.

For determining the direction as well as interrelationships between study hypotheses, SEM was employed. The goodness of fit indices was good; x2 = 495.06 (df = 183) *p* < 0.001, x2/df = 2.705, GFI = 0.901, CFI = 0.942, NFI = 0.912, RMR = 0.048, IFI = 0.942, RFI = 0.899, and RMSEA = 0.066. The results in Table 3 and Figure 3 illustrate the direct effect of hospitality work environment on WFC and turnover intention, and the indirect effect of hospitality work environment on turnover intentions through the mediating role of WFC. Hypothesis 1, which hypothesized that hospitality work environment significantly affects employees’ turnover intentions, is supported (β = 0.383, t-value = 6.340, *P* < 0.001). Moreover, hospitality work environment affects positively and significantly WFC (β = 0.528, t-value = 8.181, *P* < 0.001). Hence, H2 is supported. Hypothesis 3 which predicted that WFC significantly affects turnover intention (β = 0.219, t-value = 3.472, *P* < 0.001) is also supported. According to standardized regression analysis, behavior-based conflict followed by strain-based conflict were the higher dimensions that significantly and positively affects WFC (β = 0.862, *p* < 0.001 and β = 0.853, *p* < 0.001), respectively. In the context of the mediating role of WFC in the relationship between hospitality work environment and turnover intention, results illustrated that WFC significantly partially mediates the relationship between constructs (β = 0.116, t-value = 2.820, *P* < 0.006). As a result, H4 is supported.

**TABLE 3:** Structural parameter estimates.

Hypothesized path	Standardized path coefficients	*t*-value	Results
H_1_: Hospitality Work Environment ⟶ Turnover Intention	0.383	6.340***	Supported
H_2_: Hospitality Work Environment ⟶ WFC	0.528	8.181***	Supported
H_3_: WFC ⟶ Turnover Intention	0.219	3.472***	Supported
H_4_: Hospitality Work Environment ⟶ WFC ⟶ Turnover Intention	0.116	2.820**	Supported
Godness of fit statistics		x^2^ = 509.041 x^2^/df = 2.782 GFI = 0.901 CFI = 0.942 NFI = 0.912 RMR = 0.048 RMSEA = 0.066	

***P < 0.001, ** P < 0.01. GFI, Godness of Fit Index; CFI, Comparative Fit Index; NFI, Normed Fit Index; RMR, Root Mean Square Residual; RMSEA, Root-Mean Square Error of Approximation.

Moreover, the Sobel test is applied to assure the mediation effect of WFC on the relationship between the hospitality work environment and turnover intention. Results of the study illustrated that the raw (unstandardized) regression coefficient for the relationship between hospitality work environment and WFC was 0.316 with a standard error of 0.028. The raw coefficient for the relationship between the WFC and the employees’ turnover intention (when the hospitality work environment is also a predictor of the turnover intention) was 0.305 with a standard error of 0.077. Based on these values the test statistic for the Sobel test is calculated and produces the 3.806 (*p* < 0.001), which means that WFC significantly mediates the relationship between the hospitality work environment and employees’ turnover intention.

## Discussion and Implications

The main objective of this research study was to identify the impact of the hospitality work environment on employees’ turnover intentions and explore the potential mediating role of WFC in the relationship between the hospitality work environment and turnover intentions during the COVID-19 pandemic in a sample of three- and four-star resorts in Egyptian destinations. Upon the previous literature, the conceptual model proposed in this study hypothesized that the perceived hospitality work environment significantly affects resorts employees’ turnover intention both directly and indirectly through work-family conflict. Furthermore, the model also postulated that the hospitality work environment has a significant impact on work-family conflict, and WFC also has a significant impact on resorts’ employees’ turnover intention. Accordingly, the following findings will be discussed in literature review.

Concerning the perceptions of the investigated participants toward the hospitality work environment, it could be concluded that a higher level of negative perceptions toward the hospitality working environment during the COVID-19 pandemic has been accomplished. Irregular and unsocial working hours, followed by inappropriate working conditions and working under pressure, respectively, were the most affecting variables on their negative perceptions. These findings support the findings of Kusluvan (2003) and International Labour Organisation [ILO] (2001), who mentioned that hospitality work condition is commonly characterized by extended, unsociable, and irregular working hours in the form of weekend shifts, split shifts, nightshifts, working during the day off periods, poor physical work conditions. Furthermore, these findings are in line with that concluded by Brien et al. (2015) and Cho et al. (2009) who assured that long, irregular, and unsociable working hours, work under pressure, irregular holidays, and heavy workloads are the main characteristics of the hospitality industry.

A highly perceived WFC in all investigated dimensions has been mentioned during the COVID-19 pandemic among the investigated participants. They highly perceived that working in resorts keeps them away from their family activities more than it should be and is a frazzle to participate in family activities/responsibilities. These findings are consistent with Magnini (2009) and Blomme et al. (2010), who stated that work-family conflict is a serious human resources issue in the hospitality industry. If not managed properly, WFC can produce many detrimental consequences, including decreased employee performance, job dissatisfaction, and high turnover.

Similarly, to WFC, the investigated participants highly perceived turnover intentions. They revealed that they are thinking of resigning from my present job in the resort in the present and if they have the option to choose again, they will opt to work in another job rather than the hospitality industry. These findings support the findings concluded by previous researchers (i.e., Zeytinoglu et al., 2004; Kuria et al., 2012; AlBattat et al., 2014) who confirmed that poor hospitality work condition increases employees’ turnover intentions.

In the context of the interrelationship between the study’s constructs (hospitality work environment, WFC, and employees’ turnover intentions), it could be concluded that the hospitality work environment significantly affects employees’ turnover intentions during the COVID-19 pandemic. This result is by the findings of previous studies which confirmed that the higher perceived hospitality work environment affects directly, positively, and significantly employees’ turnover intentions. This finding is in line with the findings of Arnoux-Nicolas et al. (2016) who examined the impact of working conditions on turnover intentions of 336 French employees from diverse job contexts. The findings showed that adversative working environments (work pressure, shortage of resources, job insecurity, organizational variations, lack of individual growth, unreasonable work climate, and poor public image of the company) were significantly and positively linked with turnover intentions. In the hospitality context, unconducive work conditions, long working hours, job insecurity, split shifts, and irregular work schedule with relatively low pay were the main predictors of turnover intentions (Zeytinoglu et al., 2004; Dawson et al., 2011; Kuria et al., 2012). The results of this study are relevant to Firth et al. (2004), who concluded that a lack of supervision and support from managers in carrying out tasks will positively and significantly lead to high levels of turnover intention. On the contrary, the study’s findings are inconsistent with Kurniawaty et al. (2019) who concluded that work environment in the form of availability of supporting facilities, good physical environment, supportive management practices, and the application of good and healthy (occupational health and safety) concepts will affect significantly and negatively employees’ turnover intention.

Additionally, results revealed that the hospitality work environment had a positive and significant impact on WFC, which is in line with prior studies suggesting that the hospitality job characteristics distinctively influence WFC. Especially, employees who perceived irregular schedules and long working hours tend to ponder that work demands do not allow them to fulfill their family responsibilities (Henly et al., 2006; Zhao and Ghiselli, 2016). This finding also supports the findings of Fuß et al. (2008), who investigated the impact of working conditions on WFC among German hospital physicians, concluded that unpredictable changes in the work schedule and quantitative workload were the leading factors to increase WFC. On the other hand, this finding is inconsistent with Mesmer-Magnus and Viswesvaran (2006), who revealed that a family-friendly work environment includes flexible work arrangements, family/medical leaves, employee assistance programs, supportive work/family culture and supportive and flexible supervisors and co-workers negatively and significantly impacts WFC. Consequently, the workers who perceived a higher level of hospitality job characteristics were expected to feel heightened WFC.

Furthermore, WFC is perceived by resorts’ employees significantly and positively affects their turnover intentions, which is coincided with previous studies (i.e., Chen et al., 2018; Mansour and Tremblay, 2018; Park and Min, 2020) confirming that WFC is positively associated with turnover intentions. Moreover, this finding also supports the findings of Li et al. (2021) who examined the impact of WFC on Hong Kong police officers’ turnover intentions during COVID-19 and concluded that WFC had significant and positive effects on turnover intentions (B = 0.143, *p* < 0.05). This finding is also relevant to Yucel et al. (2021), who found a significant and positive relationship between WFC and turnover intentions during the COVID-19 outbreak among public hospital employees in Erzincan province. These findings indicate that a higher level of WFC results in a higher level of turnover intentions.

Regarding, the mediation impact of WFC on the relationship between the hospitality work environment and employees’ turnover intentions has received limited attention, specifically in the Egypt context. The findings of the present study reveal that the perceived hospitality work environment indirectly affects employees’ turnover intentions through WFC which partially mediates the relationship between study constructs. This finding is consistent with Rubel et al. (2017) who concluded that WFC plays a significant mediating role between role stressors and employees’ turnover intentions in labor-oriented organizations.

In summary, the results of this study assure that the hospitality work environment and WFC are antecedents of employees’ turnover intentions. Moreover, the perceived hospitality work environment significantly and positively affects WFC. In addition, the results show that WFC significantly mediates the relationship between the hospitality work environment and turnover intentions. Based on the study findings, the practical and theoretical implications for scholars and industry practitioners could be summarized in the following two sections.

### Theoretical Implications

The study has a few implications for researchers, specifically hospitality ones. First, the study makes a significant contribution to the general body of hypothetical literature related to the work environment, WFC, and turnover intentions in the hospitality industry specifically in the resort context. In the hospitality field, there has been limited research on the hospitality work environment, WFC, and turnover intention during the COVID-19 outbreak. In the current study, we have developed a new theoretical framework based on the TPB to examine the interrelationship between the previous constructs and determine the potential mediating role of WFC in the relationship between the hospitality work environment and turnover intentions. The proposed conceptual model contributes to work-family conflict and turnover intentions management in the hospitality industry by providing a deeper understanding of to what extent the hospitality work environment affects the attitudinal and behavioral changes of employees toward their organizations. The TPB can guide our understanding of the behavioral mechanism of the turnover intention of resorts’ employees. The study offers evidence for the direct, as well as indirect impact of the hospitality work environment on the turnover intentions of resorts’ employees. Secondly, the findings of this study could contribute to hospitality scholars in identifying the characteristics of the work environment that affect WFC and turnover intentions of resorts’ employees, which is considered an opportunity to further studies aim to explore justifications for preventing WFC and turnover intentions. Thirdly, this study showed that WFC and turnover intentions are consequences of the hospitality work environment. Hence, sufficient attention must be paid to these variables for ensuring resort employees’ retention.

### Practical Implications

In agreement with the earlier studies, this study confirmed that the hospitality work environment affects significantly WFC and employees’ turnover intentions in the era of COVID-19. Consequently, it is essential to design and implement policies and practices to reduce WFC, and turnover intention amid and after the COVID-19 pandemic. Firstly, to prevent WFC and eliminate turnover intentions, especially during unexpected emergencies like the COVID-19 pandemic, an urgent need to create a better work environment is vitally important. In prior literature, it has been documented that a comfortable working environment with adequate manpower and fair shift allocation will reduce turnover intentions (Chen and Wang, 2019). Shortage of the staff, heavy workload, lack of social support, dealing with inequality among staff, inadequate physical work settings for staff use, and a hostile work climate among colleagues should be discouraged. Second, in response to COVID-19, programs aimed at re-engaging interactions among coworkers, and establishing trust and respect, should be prioritized by HRM (McCartney et al., 2022). It is evident that creating a positive work environment, specifically resort context, is not an option for the management, but a significant aspect of work-family balance and employees’ retention. Third, workplace flexibility, including a regulated working schedule, to avoid WFC should be considered. Upon this arrangement, employees and their families could be able to organize their activities. Fourth, sufficient organizational and social support from managers and supervisors is critical. WFC serves as a mediator among hospitality work environment and turnover intentions. It means that WFC (paying attention to behavior and strain-based conflicts) can increase turnover intentions to some extent. As a result, dealing properly with employees’ needs and understanding their challenges related to WFC could help to balance family and work demands, improve their mental health, and negatively affects turnover intentions. Fifth, work-life balance initiatives should be addressed by hospitality managers. It is critically important for hospitality employers to establish a friendly workplace climate among their employees (Lee et al., 2021). Creating a family-friendly work environment that may include onsite or sponsored childcare, maternity and paternity leaves, occasional outings where families are welcome, and allowing remote work, if possible, could reduce WFC and turnover intentions.

## Limitations of the Study

This study has some limitations as follows: First, the subject of this study was resorts employees in a sample of three- and four-star resorts in Egyptian destinations. As a result, generalization of these results would be difficult. The results of this study should be applied to this specific scope of the hospitality industry. The forthcoming research might explore the perceptions of employees in other hospitality sectors. Second, this research study concentrates only on the WFC domain as a mediator in the association amid hospitality work environment and turnover intentions. Family-work conflict (FWC) as a mediator may be examined in future research. Third, the data was gathered through a web-based questionnaire, and thus, the participants could answer according to their subjective perspectives. Therefore, conducting future research using a mixed-method approach (quantitative and qualitative) may provide a better understanding. Fourth, future studies may examine the mediating role of WFC on various variables related to employees’ attitude and behavior rather than turnover intentions (satisfaction, commitment, turnover rate, etc.). Finally, the dimensions of WFC as separated mediators would be addressed.

## Data Availability Statement

The original contributions presented in the study are included in the article/supplementary material, further inquiries can be directed to the corresponding author/s.

## Ethics Statement

The studies involving human participants were reviewed and approved by Scientific Research Ethics Committee—King Faisal University. The patients/participants provided their written informed consent to participate in this study.

## Author Contributions

AA, AE, and ME: conceptualization and methodology. AA, ME, AK, and HM: software and formal analysis. AA, AK, and HM: validation and funding acquisition. ME and AK: formal analysis. AA, AE, and HM: investigation. AE and ME: data curation. AA, AE, and AK: writing—original draft. ME and HM: writing—review and editing. AA: project administration. All authors made relevant contributions and approved the final version of the manuscript.

## Conflict of Interest

The authors declare that the research was conducted in the absence of any commercial or financial relationships that could be construed as a potential conflict of interest.

## Publisher’s Note

All claims expressed in this article are solely those of the authors and do not necessarily represent those of their affiliated organizations, or those of the publisher, the editors and the reviewers. Any product that may be evaluated in this article, or claim that may be made by its manufacturer, is not guaranteed or endorsed by the publisher.
